# Endothelial dysfunction: a comprehensive appraisal

**DOI:** 10.1186/1475-2840-5-4

**Published:** 2006-02-23

**Authors:** Ricardo J Esper, Roberto A Nordaby, Jorge O Vilariño, Antonio Paragano, José L Cacharrón, Rogelio A Machado

**Affiliations:** 1 Hospital Militar Central, Departamento Cardiovascular, Servicio de Cardiología, Buenos Aires, Argentina; 2 Hospital Francés, Servicio de Cardiología, Buenos Aires, Argentina; 3 Universidad del Salvador, Escuela de Posgrado, Carrera de Cardiología, Buenos Aires, Argentina; 4 Universidad de Buenos Aires, Escuela de Medicina, Buenos Aires, Argentina; 5 Virrey Loreto 2111, C1426DXM Buenos Aires, Argentina

## Abstract

The endothelium is a thin monocelular layer that covers all the inner surface of the blood vessels, separating the circulating blood from the tissues. It is not an inactive organ, quite the opposite. It works as a receptor-efector organ and responds to each physical or chemical stimulus with the release of the correct substance with which it may maintain vasomotor balance and vascular-tissue homeostasis. It has the property of producing, independently, both agonistic and antagonistic substances that help to keep homeostasis and its function is not only autocrine, but also paracrine and endocrine. In this way it modulates the vascular smooth muscle cells producing relaxation or contraction, and therefore vasodilatation or vasoconstriction. The endothelium regulating homeostasis by controlling the production of prothrombotic and antithrombotic components, and fibrynolitics and antifibrynolitics. Also intervenes in cell proliferation and migration, in leukocyte adhesion and activation and in immunological and inflammatory processes. Cardiovascular risk factors cause oxidative stress that alters the endothelial cells capacity and leads to the so called endothelial "dysfunction" reducing its capacity to maintain homeostasis and leads to the development of pathological inflammatory processes and vascular disease.

There are different techniques to evaluate the endothelium functional capacity, that depend on the amount of NO produced and the vasodilatation effect. The percentage of vasodilatation with respect to the basal value represents the endothelial functional capacity. Taking into account that shear stress is one of the most important stimulants for the synthesis and release of NO, the non-invasive technique most often used is the transient flow-modulate "endothelium-dependent" post-ischemic vasodilatation, performed on conductance arteries such as the brachial, radial or femoral arteries. This vasodilatation is compared with the vasodilatation produced by drugs that are NO donors, such as nitroglycerine, called "endothelium independent". The vasodilatation is quantified by measuring the arterial diameter with high resolution ultrasonography. Laser-Doppler techniques are now starting to be used that also consider tissue perfusion.

There is so much proof about endothelial dysfunction that it is reasonable to believe that there is diagnostic and prognostic value in its evaluation for the late outcome. There is no doubt that endothelial dysfunction contributes to the initiation and progression of atherosclerotic disease and could be considered an independent vascular risk factor. Although prolonged randomized clinical trials are needed for unequivocal evidence, the data already obtained allows the methods of evaluation of endothelial dysfunction to be considered useful in clinical practice and have overcome the experimental step, being non-invasive increases its value making it use full for follow-up of the progression of the disease and the effects of different treatments.

## 

Ever since the endothelium was discovered by microscopical examination, it has always been considered to be a lining that acted as a barrier stopping intravascular coagulation. Nevertheless, in the last decades, the recognition of its multiple functions has shown it to be a true regulator of blood flow and tissue homeostasis. Although it is a monolayer that covers the inner surface of the entire vascular system, its total weight is more than a liver and has a mass equal to several hearts or, if it is extended, covers a various tennis courts surface area. For these reason, it has been postulated as the biggest and most important gland of the body [1].

## Anatomic and functional properties of the endothelial cell

**Figure 1:**
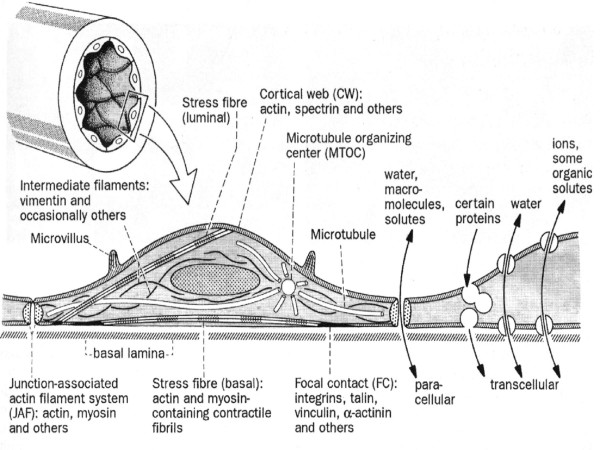
General organization of the cytoskeleton in vascular endothelial cells. From Drenckham D, Ness W.[2]

Basically, the endothelial cell has the same characteristics as all the cells of the human body; cytoplasm and organelles surrounding a nucleus and contained by the cellular membrane. The cell membrane is made of a double layer of phospholipids separated by water compartments and crossed by complex proteins that work as receptor or ion channels. Various contractile proteins cross the cytoplasm: actin, myosin, tropomyosin, α-actin and others, that allow motor activities.[2] Some are organized as structures like the cortical web, the junction-associated actin filament system related to the intercellular unions and the striated myofibril-like filament bundles or stress fibers (Figure 1).

The *cortical web *surrounds the internal surface of the sarcolema and is responsible for the cells shape and elasticity. It is sensitive to changes of the intravascular tension and increases its stiffness with increases of the intravascular pressure. It also anchor's different membrane proteins, among them annexine, which regulates endo- and exocytosis, the E-selectins and cadherine, related to the adhesiveness of leukocyte and platelets. The adherence of these elements and their passage through the endothelial cell depends on the integrity of the cortical membrane.

**Figure 2:**
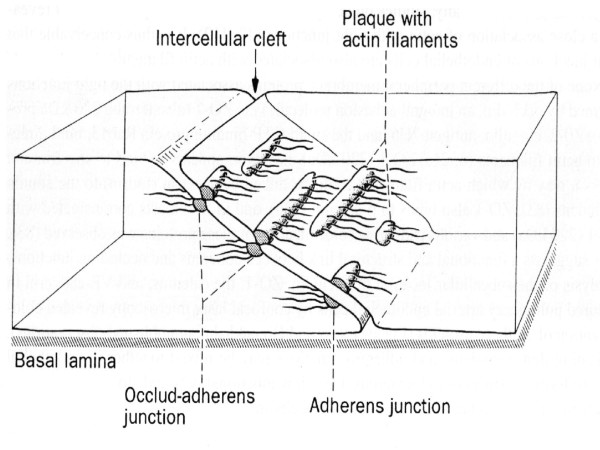
Intercellular cleft and occludens adherens junction in vascular endothelial cells. From Drenckham D, Ness W.[2]

The *junction-associated actin filament system*, known as FAU system, is found in the intercellular space and its contraction and relaxation controls the dimension of the intercellular space. In this way, it regulates the passage of solutes and macromolecules between the blood and the sub endothelial space. The Ca^2+ ^concentrations, intracellular second messenger and the common factor of the external function of cells with intermittent or cyclic activities activate it, the energy is provided by adenosine tri-phosphate (ATP). Pro-inflammatory cytokines, reactive oxygen species, thrombin, platelet activating factor, an increase of Ca^2+ ^concentration in ischemic conditions, ATP exhaustion and other toxic substances, alter the functions of the junction-associated actin filament system and allow an opening of the intercellular space and, with that, alteration of the endothelial permeability. The FAU system is closely related to the intercellular adhesion molecules, especially with VE-cadherine maintaining a balance between adhesive and contractile forces. Both cyclic adenosine mono-phosphate (cAMP), originated through the adenylate-cyclase, and the cyclic guanine mono-phosphate (cGMP), generated by a Ca^2+^-nitric oxide guanylate-cyclase dependent pathway, are second messengers that stabilize the FAU system and counteract the induction of intercellular separation, which is done through a Ca^2+^-dependent calmodulin. Nitrates, behave the same way. Protein-kinase C (PKC) activation has the opposite effect (Figure 2.)

*Stress fibers *are myofibril-like straight filament bundles composed of actin filaments interspersed with myosin filaments in a similar way as in striated muscle, and cross the cytoplasm in all directions (Figure 1). The more pressure and friction exerted by the circulating blood, the more abundant they are. As all contracting tissues, their contraction and relaxation depend on the intracellular Ca^2+ ^concentration and the presence of ATP, their principal function is to adapt the shape of the cells to the mechanical forces of blood flow and wall distention, reducing the possibility of cellular lesions. When flow increases, so does shear stress and the cells flatten and align in the direction of blood flow, whereas, when flow decreases shear stress does also, and the cells increase their volume loosing their alignment, looking like cobble stones paving.

Morphological changes acquire importance in capillary flow because they can slow or halt flow, as can be seen under the effects of serotonine, histamine, noradrenaline and trombine, although FAU also acts on this function. In the capillary the blood cells are usually larger than the diameter, but flow through the capillary by two main mechanisms: a) by the flexibility and deforming capacity of both type of cells, blood and endothelial cells, and b) by the negative electrostatic charge both cells have and, therefore, repel each other. Endothelial cells have a negative electrostatic charge because of the high concentration of sialitic acid. If this concentration is diminished for any reason, blood flow is disturbed.

The cell membrane is covered with flask-shaped membrane invaginations, sometimes shaped like a pocket and sometimes protruding out of the membrane, other times flattened, undistinguished from the basic structure of the cell membrane, but all of them very rich in lipids, sphingomyelin, complex protein structures and multiple receptors. These sites have been called "caveolae". They are so abundant that it is estimated that they occupy between 5% and 10% of the total cell surface, and are presumed to be cellular membrane receptor-efector areas.[3] In normal circumstances there are various ways of transporting plasmatic molecules through the endothelial barrier: a) intercellular unions, that generally act as filters controlled by the hydrostatic pressure that allow the passage of water and dissolved substances; b) vesicles formed from the "caveolae" that ease the passage of macromolecules through the cell membrane and cytoplasm; and c) true transcellular channels usually formed from various caveolae that connect opposite sides of the cell membrane. Through them, the endothelial regulates the passage of fluid and macromolecules between the vascular and cellular compartments, when it fails in the venous capillary area edema is produced, toxic and vasoactive substances can cause this.

## Endothelial physiology

**Figure 3:**
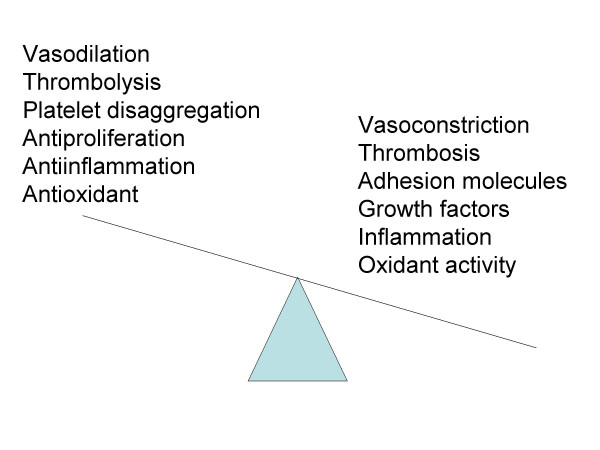
Regulatory functions of the endothelium. Normal or anti-atherogenic vs dysfunction or atherogenic propierties. From Esper RJ, et al.[5]

The endothelial cell behaves as a receptor-efector structure, that senses different physical or chemical stimulus that occur inside the vessel, and therefor modify the vessel shape or releases the necessary products to counter act the effect of the stimulus and maintain homeostasis. The endothelium is capable of producing a large variety of different molecules, as agonists as antagonists, therefore balancing the effects in both directions. Endothelium produces vasodilators and vasoconstrictors, procoagulants and anticoagulants, inflammatory and anti-inflammatory, fibrinolytics and antifibrinolytics, oxidizing and antioxidizing, and many others (Figure 3) [1,6]. When endothelial cell lose their ability to maintain this delicate balance, the conditions are right for the endothelium to be invaded by lipids and leukocytes (monocites and T-lymphocytes). The inflammatory response is incited and fatty streaks appear, the first step in the formation of the atheromatous plaque. If the situations persist, fatty streaks progress and the plaque are exposed to rupture and set the condition for thrombogenesis and the vascular occlusion.

### Nitric oxide

Nearly all stimuli that produce vasodilatation do it through nitric oxide (NO), a volatile gas, biologically active, present practically in all tissue and thanks to its low molecular weight and its lipophilic properties it diffuses easily across cell membranes. The NO crosses the endothelial intima and reaches the smooth muscular tissue of the arterial wall, and, through nitrosilation of the hem from the guanylate-cyclase degrades the GTP releasing cGMP, which in turn regulates the cytosolic Ca^2+ ^and causes smooth muscle fiber relaxation and therefore vasodilatation [7].

NO is produced by the action of nitric oxide synthases (NOS) on L-arginine aminoacid, producing NO and L-citruline, requiring O_2 _and Nicotinamide Adenine Dinucleotid Phosphate (NADP) coenzyme, essential in Redox process. Tetra-hydro-biopterine accelerates this process, which is favored by other cofactors like flavin-adenine dinucleotide, and thiol groups like cysteine and reduced-glutation. Three NOS isoenzimes are known, two constitutives and low-production, NOS-I from neurological tissue and NOS-III from endothelial cells, both respond to agonist that increase intracellular Ca^2+^. The other one, inducible NOS-II, is specially expressed in macrophage and endothelial cells due to the effect of pro-inflammatory cytokines and can release several times more NO than the constitutives NOS. Both constitutives and inducible NOS are in the endothelial cells. Constitutives NOS produces NO for short periods when it is induced by vasodilators like acetylcholine or bradikinine. Inducible NOS synthesize NO for longer periods in a constant manner when the stimulus comes from pro-inflammatory cytokines like tumor necrosis factor-α (TNF-α) [7,8].

The most important stimulation for NO release comes from shear stress, that is caused by the increase in blood velocity and leads to a vasodilatation proportional to the amount of NO released by the endothelium [9]. This vasodilatation is called endothelium-dependent. The endothelial cell membranes contains specialized ion channels, such as Ca^2+^-activated K^+ ^channels, that open in response to shear stress [10]. The effect is to hyperpolarize the endothelial cell, increasing the driving force for Ca^2+ ^entry and activates the enzyme NOS-III and the subsequent generation of NO [10]. Nitrates given in any way are NO donors, unconfined NO into the circulation directly releasing cGMP in the smooth muscle cell and causing a vasodilatation that is not dependent of the endothelial response, for this reason it is called endothelium-independent vasodilatation. Shear stress induces a persistent production of NO that maintains a constant vasodilatation [11].

Shear stress explains the importance of the haemodynamic factor in the formation, localization and plaque fissure. This usually develops in areas were the shear stress is low (<6 din/cm^2^), oscillating or retrograde, were NO release is diminished and adhesion molecules are increased and chemical and growth factors create a pro-inflammatory atmosphere. On the other hand, a high shear stress (>70 din/cm^2^) con cause endothelial erosion and provoke platelet aggregation, or cause plaque rupture or damage. That is to say that a low or retrograde shear stress allows plaque formation and progression of the atherosclerotic lesion, and a high a shear stress causes damage to the plaque. Physiological levels of shear stress that protect the endothelium are between these two values (6 to 70 din/cm2) [12-15].

NO apart of being a vasodilator, also reduces vascular permeability and the monocite and lymphocyte adhesion molecules synthesis. NO also reduces platelet aggregation, tissue oxidation, tissue inflammation, activation of thrombogenic factors, cell growth, proliferation and migration, also it inhibits proatherogenic and pro-inflammatory cytokines expression and it favors fibrinolysis. Nuclear factor kappa-B (NFkB) inhibitor (I-kB) is also expressed by NO. All these factors reduce atherogenesis and its complication. For this reason NO is considered the antiatherogenic molecule [16-22].

The endothelial cell releases angiotensin-II (A_II_) as an antagonist of NO by means of hydrolyzing angiotensin-I by angiotensine converting enzyme (ACE). Through AT_1 _receptor, A_II _causes vasoconstriction and prothrombogenic, oxidizing and antifybrinolitic effects, and also favors adhesion molecule expression and leukocyte adhesion. Also it stimulates growth and proliferation factors, activates inflammation and incite expression of pro-inflammatory and proatherogenic cytokines. All these effects allow atherosclerosis to start develop, progress and complicate. Also A_II _stimulates endothelin converting enzyme that degrades big-endothelin releasing endothelin-I, the most powerful vasoconstrictor on vessel walls [23-25].

As can be seen, depending on the balance of these two substances, NO and A_II_, a vasodilatation and antiatherosclerotic or vasoconstriction and atherogenic effect will prevail. It is not necessary by increased production of one or the other, the diminished synthesis of one, will make normal amounts of the other prevail (Figure 3). The endothelium should maintain an adequate homeostasis so that the disease does not appear and this depends on the capacity it has of producing the protective molecules. When this function is lost or damaged, we refer to it as "*endothelial dysfunction" or "endothelial activation"*. The classical cardiovascular risk factors (hypercholesterolemia, hypertension, smoking, diabetes, sedentary, etc.) and the so called new risk factors like hiperhomocysteinemia, lipoprotein Lp(a), infections by Chlamydia pneumoniae, Helicobacter Pilorae, Cytomegalovirus, herpes zoster virus or bacteroides gingivalis, all have a common factor which is a state of oxidative stress that directly or through heating proteins (HSP-60), stimulate NF-kB replication that leads to the production of proatherogenic cytokines like TNF-α, interleukins IL-1 and IL-6, adhesion molecules and chemokines, that cause inhibition of ONS-III activity and with that NO production, and favoring A_II _synthesis and activity [26-30]. The pro-inflammatory cytokines stimulate the replication of NF-kB that leads to more cytokines production. Then inflammation amplify inflammatory response [31].

It is useful to consider the other great source of vasodilatation, via arachidonic acid cascade that ends in prostaciclines, which release cAMP from ATP that regulates cytosolic Ca^2+^, therefore producing relaxation and vasodilatation. There are other vasodilatation mechanisms, like endothelium derived hyperpolarizing factor that increases the intracellular concentration of K through CP450 cytochrome, and natriuretic peptide-C intervention. It has recently been postulated that some ACE inhibitors stimulate endothelium derived hyperpolarizing factor which would explain some beneficial effects these compounds have that have not been satisfactorily explained by ACE inhibition only. All these alternative mechanisms are important in certain situations to substitute NO deficit.

### Endothelium and renin-angiotensin system

The Renin-Angiotensin System (RAS) is a cascade of enzymatic reactions that ends in A_II_. The renin, produced by the kidney, acts on the angiotensinogen, produced by the liver degrading it to A_I_, which in turn will be acted on by circulating or tissue ACE and hydrolyzed to A_II_. Chymase, Carboxypeptidase, Cathepsin G and Tonin can generate A_II _from A_I _independently of ACE, and A_II _can also originate directly from angiotensinogen by non-renin enzymes, such as tissue plasminogen activator (t-AP), Cathepsin G and Tonin. Because of structural similarity ACE also degrades other peptides that are substrates to this enzyme such as P substance, enkephalins, neurotensin, takynine and kininogen, the latter responsible for bradykinin generation. Stimulates ONS-III, inducing ON synthesis and prostacicline production, which in turn have opposite the effects of A_II_. As can be seen, the interrelationship between agonists and antagonists is very complex and requires a delicate balance which is the endotheliums primary function [26-31]. There are two RAS, one is circulatory and the other is in the tissues, the latter at a cellular level develops approximately 90% of the systems activity.

A_II _acts by stimulating specific receptors, in the human body we are able to distinguish two different types of receptors: AT_1 _and AT_2_. the AT_1 _receptors are responsible for known effects of A_II_; vasoconstriction, increase of aldosterone activity, myocardial hypertrophy, vessel wall smooth muscle proliferation, renal sodium reabsortion, increase in peripheral noradrenergic activity, vasopresin release, sympathetic stimulation, decrease in renal blood flow, etc. The effects of AT_2 _receptor stimulation is not completely known, but there are animal and human data that allow us to assume that they era responsible for apoptosis, a clear inhibition of proliferation, vascular endothelial neogenesis stimulation and vasodilatation, all opposite effects of AT_1 _[32]. AT_2 _receptors are found during the last three months of pregnancy in the fetus, and during the first three weeks of life. In adult tissues, they are predominantly expressed in the brain and adrenals, with lower levels expressed elsewhere [33]. They are expressed when there is vascular injury.

There are different angiotensine peptides capable of stimulating the AT_1 _receptors with varying intensity: A_II_(2–8), A_IV_(3–8), A_II_(1–7). This fact and the different effects of each one when the specific receptors were blocked suggest that there are other specific receptors sensitive to each of these peptides. In experimental animal models different specific receptor variant have been isolated and cloned, for example in the rat AT_1A_, AT_1B_, and AT_1C_. Nevertheless, in human beings, only AT_1 _and AT_2 _receptors of AT_II _have been identified. A_II_(1–7) has opposite effects of the classic A_II_, producing vasodilatation, inhibiting proliferation, myocardial hypertrophy and vascular smooth muscle proliferation, and it is very unlikely that it should act through the known AT_1 _and AT_2 _receptors. This led to believe that there are specific receptors for it. This has actually been reported recently although they have not yet been cloned [32-34].

## Oxidant byproducts and atherosclerosis

Oxidant products, such as superoxide anion (O_2_^-^), hydrogen peroxide (H_2_O_2_), hydroxyl radical (HO), hypochlorous acid (HOCl) and lipid radicals [33], are produced as a consequence of normal aerobic metabolism. These molecules are highly reactive with other biological molecules and are referred as Reactive Oxygen Species (ROS). Under normal physiological conditions, ROS production is balanced by an efficient system of antioxidants, molecules that are capable to neutralize them and thereby preventing oxidant damage. In the tissues, naturally occurring enzymatic antioxidants such as superoxide dismutase, glutathione peroxidase, and catalase play an important role in the conversion of ROS to oxygen and water. Several nonenzymatic antioxidants, including the lipid-soluble vitamin E and β-carotene and the water-soluble antioxidants vitamin C, which particular protects plasma lipids from peroxidation, scavenges superoxide anion, and plays a role in recycling vitamin E [35]. In pathological states, the ROS may be present in relative excess. This shift of the balance in favor of oxidation termed "oxidative stress", may have detrimental effects on cellular and tissue function. As mentioned before, cardiovascular risk factors generate oxidative stress (Table I).

**Table 1:** Normal aerobic metabolism oxidizing and antioxidizing systems

**OXIDIZING SYSTEMS**	**ANTIOXIDIZING SYSTEMS**
**Oxidant byproducts**	**Enzymatic antioxidants**
Superoxide Anion (O_2_^-^)	Superoxide dismutase
Hydrogen Peroxide (H_2 _O_2_)	Glutathione peroxidase
Hydroxyl radical (HO)	Catalase
Hypochlorous acid (HOCl)	
Lipid radicals	
	
**Enzimatic oxidants**	**Lipid-soluble antioxidants**
Dehidrogenase Xantine	Vitamin E
NADPH oxidase	β-Carotene
Mieloperoxidase	
Monoamino oxidase	**Water-soluble antioxidants**
ON synthetase	Vitamin C

LDL-cholesterol molecules are easily oxidized in a state of oxidative stress, especially the small and dense molecules. Native LDL molecules are innocuous, they do not produce any inflammatory reaction and do not lead to foam cells when phagocyte by specific native macrophage receptors. Oxidized LDL-cholesterol molecules (LDL-ox), are highly immunogenic, and is associated with up-regulation of pattern-recognition receptors for innate immunity, including scavengers receptors and toll-like receptors [36,37]. They are found in all atherosclerotic lesions and they generate antibodies that are capable of neutralizing them. LDL-ox attack the arterial intima and leads to release of phospholipids that can activate endothelial cells [38], induces the production of endothelium adhesion molecules and monocytes attraction [39], has endothelium cytotoxic effect, increases proinflamatory gene activity and cellular growth factors, it provokes endothelial dysfunction, platelet aggregation, metaloproteinase expression and favors thrombogenesis [40]. LDL-ox molecules are found in the subendothelial layers and help to activate monocytes that are transformed into macrophage, upregulating their scavenger receptors and toll-like receptors that then phagocyte them. With progressive accumulation of LDL-ox macrophage modulate their phenotype turning them into foam cells. Foam cells are the principal component of the fatty streaks, first step in atheromatous plaque formation, and they trigger antigenic reaction in T-lymphocytes that initiate or increase the immunological response [41]. Also TNF-α is activated and endothelial cell apoptosis is induced and has a close relation with the severity of acute ischaemic syndromes [42,43].

HDL cholesterol and apolipoprotein A-1 have direct antiatherogenic and vascular protective effects. They have antioxidant effects attributed to the binding of transition metals and to the presence of paraoxonase, an enzyme carried predominantely by apolipoproteins A-1 and J, containing HDL particles, which has powerful antioxidant effects. Morover, they have been shown anti-inflammatory effects, scavenging of toxic phospholipids, stimulation of reverse cholesterol transport, antithrombotic and profibrinolytic effects, and attenuation of endothelial dysfunction [44].

The excess of ROS, especially superoxide anion, can oxidize NO and transform it into peroxynitrite (ONOO), a inactive molecule that can lead to more oxidation. This situation is usually seen when ONS-II activation is induced by the high concentration of NO it generates. This also happens with high levels of LDL, especially small and dense molecules that are prone to oxidation. Asymmetric dimethyl-amino-arginine (ADMA) exists normally in the body and it inhibits NO synthesis by competing with L-arginine. In this way, reduce NO tissue concentration with all the consequences that this causes, to the extent that many investigators consider it a new atherosclerotic risk factor. Serum levels of ADMA keep a close relationship with LDL-ox concentration and viceversa [45,46]. Peroxinitrite (ONOO) can oxidize tetra-hidro-biopterin, a critical cofactor for ONS [47].

Long-term with most organic nitrates is frequently associated with a progressive reduction of hemodynamic effects. Nitrates activates vascular NADPH oxidase with incremental O_2_^- ^generations, and this highly reactive molecules oxidize NO to ONOO [48]. Moreover, continuos treatment with nitrates causes ONS-III dysfunction by oxidative stress. Reduced bioavailability of tetra-hydro-biopterin is involved in the pathogenesis of this phenomenon and is prevented by supplemental folic acid administration. ROS transform regular ONS-III function, and produces O_2_^- ^in place of NO [49]. Oxidative stress may explain the development of tolerance and the impaired endothelial function during continuous organic nitrates administration.

## Inflammation and thrombosis

There is a clear relationship between inflammation and thrombosis, each influencing the other. Inflammatory cytokines induce procoagulant molecules in endothelial cells such as von Willebrand factor, tissue factor and plasminogen activating inhibitor factors, PAI-1 and PAI-2. Activated inflammatory cells also produce molecules that contribute to the thrombogenesis such as tissue factor and Thrombin, which in turn generates an intense mitogenic stimulus and platelet activation [50-52]. Interleukin-6 not only increase plasma concentration of C-Reactive Protein (CRP) in the liver but also fibrinogen, PAI-1, and serum-A amyloid protein. On the other hand, CRP amplify immunological response inducing leukocyte adhesion molecules and chemokines production in the endothelial cells, and show synergetic action with bacterial polysaccharides inducing monocyte grow factors [53]. Interleuquine IL-1 provokes synthesis of PAI-1 in the endothelial cells whereas IL-4 induces plasminogen tissue activator (t-PA) by monocytes. Cell surface-based signaling system CD40L ligand (CD154), binding to its receptor CD40 on the leucocyte, can induce tissue factor expression [50]. Platelets can express CD154, the molecule that regulates tissue factor gene expression in the macrophage and smooth muscle cells [51,52]. As can be seen, this process is a complex feed-back in which inflammation favors thrombosis, anti-inflammatory treatment has antithrombotic effects and viceversa [54,55].

## Immunological system and atherogenesis

Atherosclerosis is related to activation of the immunological system [56]. The developing atherosclerotic plaque are infiltrated not only by macrophage but by T-lymphocyte (CD-4)Th called helper, and T-lymphocyte CD-8, that suggest a specific immunological response [57]. Never the less investigators have not yet reached an agreement on whether the effect is harmful or beneficial on the developing plaque. T-lymphocytes (CD4)Th1 produce TNF-α, Interferon-γ and IL-6, are all pro-inflammatory compounds that activate macrophage and are responsible for the late hypersensitivity reactions. On the other hand, T-lymphocytes (CD-4)Th2 generate IL-4, IL-5, IL-10 and IL-13, all of these anti-inflammatory molecules that promote antibody responses and energetically inhibit macrophage activity [58]. In the atherosclerotic plaque of experimental animals and in human beings, inflammatory cytokines produced by T-lymphocyte (CD4)Th1, such as Interferon-γ and IL-12, have been found in a pro-inflammatory surrounding similar to reumathoid arthritis [59,62]. In other plaques they have not been detected, so it is suspected that there is a reduction in the inflammatory response which leads to the believe that the balance between T-lymphocytes (CD-4)Th1 and Th2, may play an important role in the progression or regression of the plaque. In this respect statins play a role modulating immunological activity [63-65].

Sphingomyelinase is another immunological mediator produced by macrophage and endothelial cell when stimulated by inflammatory cytokines. It is one of the substances that are responsible for oxidized lipoproteins passing through the endothelium, for foam cells formation and progression, and complication of the atherosclerotic plaque [66,67].

## From endothelial dysfunction to acute ischemic syndrome

Atherosclerosis has been considered as a disease of "*four concepts*". In a substudy of 5.209 patients from the Framingham Study followed for 10 years, it was seen that those patients with peripheral vascular disease had more probabilities of having an Acute Myocardial Infarction (AMI) or a Stroke, while those who had had an AMI had more possibilities of having a Stroke or peripheral vascular disease. Also those who had suffered a Stroke had more chances of having an AMI or peripheral vascular disease. From these observation the first concept, that *atherosclerosis is a diffuse disease*, was postulated [68,69].

The finding of lesions in different stages of development throughout the body, and inclusive in the same territory, lead to the second concept that "*atherosclerosis is a heterogeneous and multiform disease" *[69].

In advanced stages of arteriosclerosis, with stage IV and Va by AHA classification or Ross type III lesion, two types of lesions can be distinguished: I) Stable or fibrous plaque, with a small and generally central lipid core protected by a thick and resistant cover with a high content of collagen and without signs of inflammation. These lesions usually obstruct the vessel significantly and are easily seen by arteriography. II) High Risk, unstable or vulnerable plaque, with a large lipid core usually eccentric, covered by a weak and thin fibrous cap with little collagen and large quantities of macrophage and T-lymphocytes that are expression of a great inflammatory reaction that seldomly occlude the vessel significantly and is frequently not appreciated by angiography. This has generated the third concept that the "*quality of the plaque is more important than the size"*, as proved by the fact that these plaques rupture easily and are responsible for the majority of the acute coronary syndromes. According to a metaanalysis by Falk et al, these high risk plaque which obstruct less than 70% and even less than 50% usually are asymptomatic, they are not easily recognized and not considered significant by angiography are responsible for 86% of acute coronary syndromes [69-72].

In atherosclerotic disease classical risk factors play an important part; high cholesterol serum levels, arterial hypertension, smoking, obesity, sedentariness, and the so called new risk factors such as hyperhomocysteinemia, Lp(a) lipoprotein, Cytomegalovirus, Chlamydia pneumoniae, Helicobacter pilorae, Bacteroides gingivalis, genetic factors (gene ECA, gene HLA and others), serum inflammatory markers (CRP, serum-A amyloid protein, and others), and prothrombotic factors (PAI-I, D-dimer, fibrinogen, von Willebrand, etc.), microalbumineamia, all of these factors contribute, in different degrees, to make acute coronary syndromes happen. This suggests the fourth concept, that "*atherosclerosis is an inflammatory, immunological, polygenic and multifactorial disease" *[72-77].

The evolution of the disease can be slow and patients with risk factors develop chronic or unstable angina or AMI, this form is observed in less than 40%, or it can develop abruptly in patients who had low risk and were asymptomatic when they develop unstable angina, AMI or sudden death. This is found in more than 60% of patients and this fact obliges us to find another explanation based on a new physiopathological model.

All the experience obtained in the last years, suggests that the endothelium's dysfunction is no only the initial stage of the atherosclerotic disease that generates plaque formation, but also can causes plaque growth, and unprotected the high risk plaque leading to develop a vascular event. Between these two extremes endothelial dysfunction is responsible for all the plaque growth, differences in plaque development and plaque characteristics. For all these reasons, endothelial dysfunction is one of the principal mechanisms in atherosclerotic disease [78]. The presence of classical and new risk factors generates a chronic exogenous state of injury to the endothelium that promotes abnormal response, vasoconstriction, accumulation of inflammatory cells, migration of smooth muscle cells, increased cytokine production, etc, all these factors help atheromatous plaque formation and in turn generate a negative feedback that leads to a second injury, this time endogenous, that finally leaves the plaque unprotected allowing it to rupture or erosion and triggering thrombogenic phenomena [79].

## Diabetes and endothelium

Both type 1 (insulin-dependent) and type 2 (no insulin-dependent) diabetic patients, have mostly been described under enhanced oxidative stress, and both conditions are known to be powerful and independent risk factors for coronary heart disease, stroke, and peripheral arterial disease.

Hyperglycemia causes glycosylation of proteins and phospholipids, thus increasing intracellular oxidative stress. Nonenzymatic reactive products, known as Maillard or browning reaction, glucose-derived Schiff base, and Amadori products, form chemically reversible early glycosylation products which subsequently rearrange to form more stable products, some of them on long-lived proteins (e.g. vessel wall collagen) and continue undergoing complex series of chemical rearrangements to form advanced glycosylation end products (AGEs). Once formed, AGEs are stable and virtually irreversible. AGEs generate ROS with consequent increased vessel oxidative damage [80].

Phagocytes have specialized receptors for AGEs, their activation leading to oxidation of lipoproteins, especially the phospholipid component in LDL, and stimulating an immune-inflamatory response and a thrombogenic response through Tromboxane A_2 _release and platelet aggregation induction. Diabetic patients have increased levels of inflammatory markers, including CRP, with proinflammatory and proatherogenic properties.

The impressive correlation between coronary artery disease and alterations in glucose metabolism has raised the hypotesis that atherosclerosis and diabetes may share common antecedents. Large-vessel atherosclerosis can precede the development of diabetes, suggesting that rather than atherosclerosis being a complication of diabetes, both conditions may share genetic and environmental antecedents, a "common soil" [81].

Published data suggest that abnormal endothelial function precedes other evidence of vascular disease and that the progresion of metabolic syndrome to type 2 diabetes parallels the progression of endothelial dysfunction to atherosclerosis. Both type 1 and type 2 diabetes, like metabolic syndrome and other cardiovascular risk factors determine an abnormal endothelium response thought to precede the development of atherosclerosis.

### Lipoprotein-associated phospholipase A_2 _(LP PLA2)

The lipoprotein-associated phospholipase A_2 _(LP PLA2), alternatively termed as platelet activating factor acetylhydrolase, is a calcium-independent phospholipase which hydrolyzes specifically the short acyl groups at the sn-2 position of the phospholipid substrate. Thus LP PLA2 plays a key role in the degradation of proinflammatory oxidized phospholipids (oxPL) and in the generation of lysophosphatidylcholine (lyso-PC) and oxidized fatty acids. LP PLA2 can also hydrolyze short-chain diacylglycerols, triacylglycerols, and acetylated alkanols, and displays a PLA1 activity [82].

The LP PLA2 has been considered a risk marker for endothelial dysfunction in diabetic patients. LP PLA2 levels have show a significant and positive correlation with CRP levels, reflecting the possible relation of LP PLA2 with inflammatory activity in atherosclerotic arteries [83]. Packard et al confirmed this previous data in a substudy of the West of Scotland Coronary Prevention Study in which they compared different inflammatory markers as cardiovascular risk predictors [84]. They found that in these patients LP PLA2 levels had a strong, positive association with the development of coronary events i.e., myocardial infaction, cardiovascular death or revascularization procedure, which was not confounded by other risk factors. The patients in which LP PLA2 levels were in the highest quintile had almost twice the risk of those in whom they were in the lowest quintile [85].

Myeloperoxidase and Paraoxonase are other enzymes involved in the evolution of endothelial dysfunction associated with type-2 diabetes [86].

## Erectile dysfunction, sildenafil and endothelial function

Erectile dysfunction is an important complication of cardiovsascular disease and can work as a marker of endothelial function as well as a predictor of elevated risk of cardiovascular disease. About 30% of diabetic patients are afected by it [87].

Ischaemic heart disease can contribute to erectile dysfunction usually through fear although it can also be the first expression of coronary heart disease. Endothelial dysfunction is often found in patients with erectile dysfunction.

Erection is produced when erotic stimuli are percived by diferent senses and reach the hypothalamus thus inhibiting the simpathetic tone and releasing NO at the non adrenergic and non colinergic nerve terminals and in the endothelial cells of the penile arterioles. The NO reaches the cavernous smooth muscle and the arteriolar wall activating the enzime guanylate cyclase that degrades GTP releasing GMP, which reduces the smooth muscle Ca^2+ ^uptake and produces vasodilatation. Phosphodiesterase-5 (PDE-5) reverts this situation by changing GMP back to GTP. This enzime is found in the corpus cavernosum, blood vessels and trachea, but not in the myocardium, and is inhibited by sildenafil, that in this way prolongs erection. Sildenafil is very highly selective for PDE-5 compared to other PDE, so as to be considered to have a PDE-5/PDE-3 selectivity ratio equal to 4000:1. This fact does not allow sildenafil to have a measurable positive inotropic effect on the myocardium [88].

## Assessment endothelial function. Invasive and non-invasive techniques

Endothelial dysfunction, or loss or reduction of its capacity of defense against proatherogenic factors, is obtained evaluating any of the endothelium functions. For example, quantifying circulating adhesion molecules, proatherogenic substances, antifibrinolytics, evaluation of serum markers of inflammation, etc. All these are direct or indirect markers the endothelium capacity to protect against new atherosclerotic lesions or protect existing lesions from causing a vascular event. Because of the ease with which it can be done and its reliability, the most commonly used test in basic science and clinical research is endothelial dependent vasodilatation modulated by flow, considered at this moment the gold standard [78].

The first experiments to evaluate endothelial dependent vasodilatation were performed by invasive techniques *catheterizing coronary arteries*, were drugs that induced NO release were injected, such as acetylcholine, metacholine, papaverine, P substance, etc, and then measuring the percentage of vasodilatation. Ludmer et al [11], found that injecting acetylcholine in normal coronary arteries produced endothelial dependent vasodilatation, were as in coronary arteries with moderate or severe atherosclerotic lesions a paradoxical vasoconstriction was obtained, indicating that endothelial dysfunction was present. The paradoxical vasoconstriction is due to stimulation of the muscarinic receptors of the smooth muscle cells by direct acetylcholine action. It was also found that injecting nitroglycerine, an NO donor, there was always vasodilatation, in this case considered endothelium independent vasodilatation. Vita et al [88], using the same method found that the amount of coronary vasodilatation obtained by acetylcholine diminished in an inverse ratio with the increase of total cholesterol or LDL-cholesterol levels. They also observed that the presence of cardiovascular risk factors, alone or combined, kept an inverse linear relationship with the endothelium dependent vasodilatation response, evidencing the additive effects on endothelial dysfunction [88].

Later *forearm pletismography *was used. This technique could be considered partially invasive. It was performed by placing the forearm in a pletismograph for veins impedance and then injects the drug being studied, usually acetylcholine or metacholine, into the brachial artery. Panza et al [89-91], studying hypertensive patients, found them to have less vasodilatation response than normal controls. Also, hypertensive patients had an increase in vascular resistance in comparison with normotensive patients, which was permitted by the endothelial dysfunction. As endothelium maintains a constant vasodilatation level produced by NO, it was considered that in hypertension there was an endothelial dysfunction that reduced NO release, and with that a reduced basal vasodilatation. This led them to postulate that endothelial dysfunction could be one of the causes of hypertension. Later on, other working groups concluded that endothelial dysfunction was probably a consequence and not the cause of hypertension [88-91]. This technique mainly evaluates the resistance arteries response.

**Figure 4:**
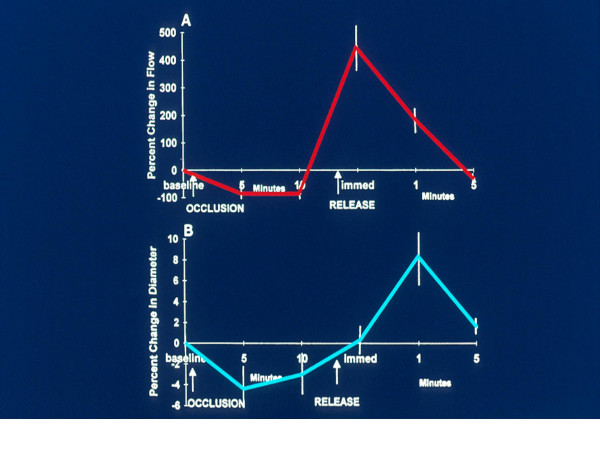
Flow mediated post-ischemic endothelial dependent vasodilatation. Percent change in flow and diameter after occlusion. From Correti MC, et al.[93]

In the last years, Celermajer et al [92], assessed the level of vasodilatation by ultrasonography. This technique, non-invasive and easy to repeat, by which due to the changes that occur through time allow us to learn the natural evolution of the disease or see the changes produced by the different treatments given. Consists in causing forearm ischaemia and observes the amount of post ischaemic vasodilatation. Ischaemia is produced by compressing the forearm by inflating a conventional cuff for measuring blood pressure 30 mmHg above systolic pressure for five minutes. When the pressure is released there is a marked increase of flow to the forearm and this increases shear stress which in turn stimulates NO release that causes vasodilatation. The amount of vasodilatation is directly proportional to the amount of NO released by the endothelium and this allows us to evaluate endothelial function. The increase in flow and vasodilatation is measured by high-resolution ultrasonography of the brachial artery, and is expressed as a percentage of the increase of the basal values [93,94]. It can also be performed on the radial and femoral arteries [92,94]. This technique evaluates mainly the conductance vessels, different from pletismography that mainly evaluates the resistance vessels, although both methods test NO release (Figure 4).

**Figure 5:**
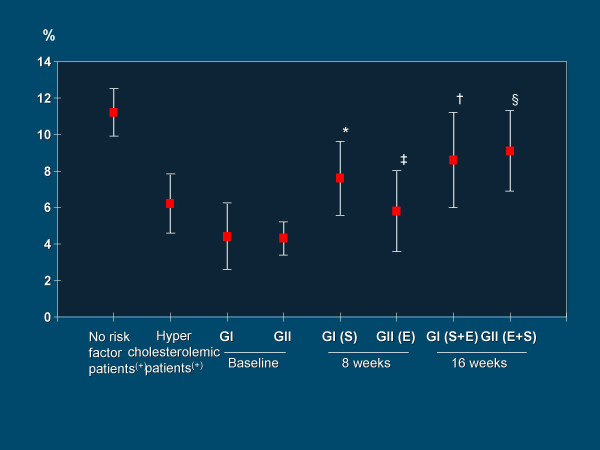
Flow mediated post-ischemic endothelium-dependent vasodilatation expressed as percent increase in arterial diameter (mean ± SD) with respect to baseline values in hypercholesterolemic coronary artery disease patients under the effects of the statin Simvastatin and the ECA inhibitor Enalapril, either separately or combined. **E**: Enalapril, **GI**: Group I, **GII**: Group II, **S**: Simvastatin. * p < 0.001 vs baseline, ‡ p < 0.01 vs Baseline, † p < 0.05 vs 8 weeks, § p < 0.001 vs 8 weeks. From Esper RJ, et al.[131]

This method has shown a gradual decrease in the endothelium dependent response depending on age, due to gradual loss of NO synthesis by the endothelial cells [95-97]. This same response is seen in patients with atherosclerotic lesions whether symptomatic or asymptomatic [97-99], in post-menopause women due to lack of estrogen [100,101], and in presence of cardiovascular risk factor such as hypercholesterolemia [102], hypertension [103], active smoking [104,105], passive smoking [106], obesity [107], diabetes [108], sedentariness [109], and hyperhomocysteinemia [110]. Mild infections [111] and increasing levels of CPR may also decrease de endothelial response [112]. The reversal of the cardiovascular risk factors allows a better endothelial function increasing the endothelial dependent vasodilatation which expresses an increase in NO release. The reduction of serum cholesterol levels [113-116], arterial pressure control [103,113], quit smoking [94], weight control [107], diabetes improvement [118], and physical activity [119] improving the endothelial dependent response, indicating more ON released. The administration of L-arginine, a NO precursor, increases vasodilatation in patients with high cholesterol, coronary artery disease and heart failure [120-122]. As has been explained previously, all these risk factors act by a common pathway, oxidative stress, with increased production of ROS [124,125]. It has been observed that giving anti oxidant vitamins C and E diminishes the production of ROS [126-128], including when given before a meal with a high fat content to reduce oxidation of post absorption fatty acids and tryglicerydes [129], it is also effective before smoking [130]. Recently it has been reported that there is an additive effect with the simultaneous administration of a statin and ACE inhibitor in hipercholesterolemic coronary artery disease patients (Figure 5) [131].

The National Cholesterol Education Program (NCEP) recommends a maximum LDL cholesterol level of 160 mg/dl in healthy population without risk factors, 130 mg/dl for primary prevention, 100 mg/dl for secondary prevention and 70 mg/dl for high risk patients. Nevertheless, Steinberg et al [132], have evaluated endothelial function in healthy young patients with this technique without cardiovascular risk factors and cholesterol levels between 146 and 195 mg/dl and LDL-cholesterol between 87 and 119 mg/dl, and found that the lower the total cholesterol and LDL cholesterol levels, the better the vasodilatation response, as an expression of better endothelial response, which leads to the idea that the lower the cholesterol the better the endothelial response [114].

Although the method of evaluating endothelial response by measurements on the brachial artery is non-invasive and easily repeated, without any risk for the patient, it is time consuming and needs skilled and patient operators [78]. With just a small displacement of the transducer the results are altered. Some automated systems have shown a reduction in the variation between operators [78,133,134]. Some investigators have developed different equipment to maintain the transducer in a constant position. It is a well known fact that the response is different whether the cuff is positioned on the arm or forearm. Recently, an International task force published the guidelines for the techniques and assessment of this method [94].

Looking for simpler methods of performing these procedures laser-Doppler has recently been used. This technique considers the vasodilatation and the increase in blood flow, evaluation tissue perfusion. It has the advantage of being simple with immediate results and does not need skilled operators [135,136].

## Prognostic value of post-ischaemic flow mediated endothelium dependent vasodilatation

Although a lot has been published about post-ischemic flow mediated endothelium dependent vasodilatation as a test for endothelial function the same doubts remain: can we compare the brachial artery with the coronary artery?, and, does this technique have a prognostic value?.

The endothelial function of the brachial artery measured by this method has shown a good correlation with the coronary artery response at the same time and in the same patient with acetylcholine and with other methods which has led to consider the brachial artery as a "surrogate" of the coronary artery [137,138]. It has also been shown at necropsy that patients with grade I-III lesions in the left descending coronary artery, had lesions of the same type and severity in their brachial artery, confirming the similarity [138]. Respect the sensibility of the method, using 7.5 Mhz or more transducers, modest changes of 0,1 mm can be measured precisely and are easily reproduced [78,94]. It has been shown that the inter-observer variation, with qualified observers is 0,1 mm [78,94].

With respect of the prognostic value, as this is a new method, not enough time has elapsed for a proper evaluation [139]. Never the less, an excellent correlation has been noted with the severity of the vascular lesions, even when they are asymptomatic and undetectable by the usual clinical methods. It has also been reported that patients without cardiovascular disease but with risk factors, had a brachial artery vasodilatation response diminished when compared with a normal population, and if they had a peripheral artery disease the response was even more diminished compared with the positive risk factor population. This leads to the assumption that as the severity of the lesions increases, so does the endothelial dysfunction [140].

Some authors have related left ventricular mass with endothelial function, and have reported that with normal ventricular geometry, endothelial function was within normal limits, were as in patients with concentric remodeling of the left ventricle showed a depressed endothelial function, and in patients with concentric or eccentric left ventricular hypertrophy the decrease of endothelial function was even greater [141]. The functional class by stress test also has a close relation with endothelial function. Those patients who reached 100 to 150 watts had a normal endothelial dependent vasodilatation similar to controls, but if they reached 75 or less watts, the vasodilatation response was depressed. The response to nitroglycerine showed a similar response in both groups.

Schachinger, et. al. [142] in an average of 82 month follow up, documented 14 major cardiovascular events, sudden death, AMI, unstable angina, stroke, or revascularization procedures, in patients in the inferior tertile of the vasodilatation response, and found 5 events in the superior tertile of the population. Both the endothelial dependent and endothelial independent response and their ratio (EDV/EIV) were strongly related to future cardiovascular events. Neunteufl et. al. [143] in a long follow up, found that the depressed vasodilatation response can predict the cardiovascular event risk for the next 5 years. In patient with normal vasodilatation versus abnormal vasodilatation, with a cut off at >10% in normal and <10% in abnormal response, the need for PTCA or CABG differed significantly, with a greater tendency of AMI in the patients with a poor response. Considering the total event rates, the flow mediated post-ischaemic endothelial vasodilatation response measured by high resolution ultrasonography of the brachial artery showed a sensitivity of 86% with a 51% specificity and a very important negative predictive value of 93%.

Al Suwaidi et. al. [144] studied patients with mild coronary artery disease (<30% stenosis) with quantitative coronary angiography with a follow up of 8 months, and found that patients with severe endothelial dysfunction with acetylcholine had a significant increase of events, 14% when compared to patients with a normal or mildly depressed vasodilatation response.

Schoeder et. al. [145] compared post ischaemic vasodilatation response of the brachial artery with the predictive value of angina, stress test and radioisotope myocardial perfusion. They found that this test had a sensitivity of 71%, a specificity of 81% and a positive predictive value of 95%, similar values to the other three diagnostic tests.

**Figure 6:**
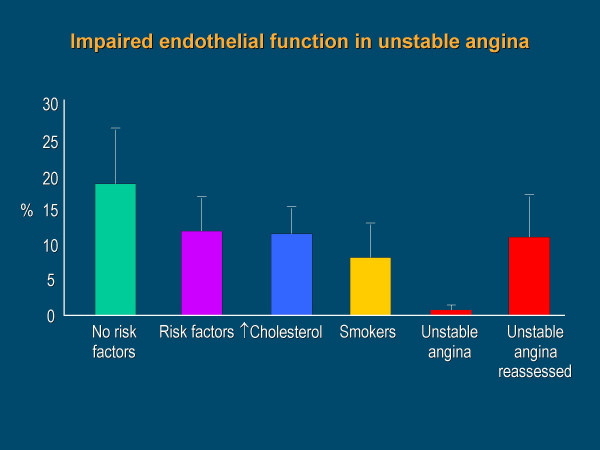
Endothelium depended vasodilatation in no cardiovascular risk factor subjects and in patients with risk factors, hypercholesterolemia, smokers, within 24 hours of rapidly stabilized unstable angina, and approximately 30 days later, after pharmacological treatment. From Esper RJ, et al.[146]

Our group [146], found that endothelial dependant vasodilatation during unstable angina is practically absent, creating a doubt about whether "the endothelial dysfunction is caused by the plaque rupture or if it existed previously and did not protect the high risk atheromatous plaque allowing the acute coronary event". Based on this study and comparing with what happens to the ischaemic myocardium, created the concept of "stunned" endothelium to explain the low vasodilatation response in acute ischaemia and "hibernated" endothelium when the poor response is chronic, as observed with hypercholesterolemia and the presence of other risk factor (Figure 6).

Cohen et. al. [147] in a subgroup of patients taking part in the CARE trial, studied the brachial artery vasodilatation with ultrasound and found that post AMI patients treated with statins and with cholesterol values considered "normal", had a significantly greater vasodilatation than the group that received placebo. The magnitude of the increase was correlated with the decrease of the LDL cholesterol fraction. It has been considered that the improvement of the vasodilatation response could be the main mechanism of the reduction of recurrent events found in the CARE trial.

It has been reported that non-invasive assessment of endothelial function in the brachial artery predicts perioperative cardiovascular events in patients undergoing vascular surgery [148]. In 1479 subjects attending a routine exam at the Framingham Study, it has been shown that advanced age and systolic blood pressure are strongly associated with decreased endothelial function, and the dilatation was larger in females vs males [149].

Ganz et al. discussed the pronostic value of the post-ischaemic flow mediated endotelial vasodilatation in a revision of the last 10 trials on the subject, and concluded that is a useful diagnosis procedure with a high pronostic value. In the same issue, in editorials by Verma et al, and Willerson et al., arrived at similar conclusions [150-152].

The atherosclerotic lesion always starts with endothelial dysfunction and progresses with the persistence of this dysfunction [78,153-157]. From a practical point of view, the established vascular lesions seen by angiography, Intravascular Ultrasound (IVUS), Magnetic Nuclear Resonance, Computed bean Tomography, Carotid ultrasonography, etc, could be considered like the electrocardiogram in ischemic heart disease, were as the techniques for endothelial function evaluation would be similar to the conclusions reached with stress tests, reassuring the hypothesis that functional alterations precede the anatomic lesions in the development of atherosclerosis and the ischaemic cascade [156,157]. This idea is reinforced by the relation between endothelial dysfunction and microalbuminuria, another risk marker that is not often taken into consideration [158].
